# The origin of RNA interference: Adaptive or neutral evolution?

**DOI:** 10.1371/journal.pbio.3001715

**Published:** 2022-06-29

**Authors:** Alessandro Torri, Johannes Jaeger, Thomas Pradeu, Maria-Carla Saleh

**Affiliations:** 1 Virus & RNA interference Unit, Department of Virology, Institut Pasteur, CNRS UMR 3569, Université Paris Cité, Paris, France; 2 Complexity Science Hub (CSH) Vienna, Vienna, Austria; 3 ImmunoConcEpT, CNRS UMR 5164, University of Bordeaux, Bordeaux, France; 4 Institut d’histoire et de philosophie des sciences et des techniques, CNRS UMR 8590, Pantheon-Sorbonne University, Paris, France

## Abstract

The origin of RNA interference (RNAi) is usually explained by a defense-based hypothesis, in which RNAi evolved as a defense against transposable elements (TEs) and RNA viruses and was already present in the last eukaryotic common ancestor (LECA). However, since RNA antisense regulation and double-stranded RNAs (dsRNAs) are ancient and widespread phenomena, the origin of defensive RNAi should have occurred in parallel with its regulative functions to avoid imbalances in gene regulation. Thus, we propose a neutral evolutionary hypothesis for the origin of RNAi in which qualitative system drift from a prokaryotic antisense RNA gene regulation mechanism leads to the formation of RNAi through constructive neutral evolution (CNE). We argue that RNAi was already present in the ancestor of LECA before the need for a new defense system arose and that its presence helped to shape eukaryotic genomic architecture and stability.

## Abstract 2

Where does RNA interference come from? This Essay describes a new step-by-step evolutionary model of how RNA interference might have originated in early eukaryotes through neutral events from the molecular machinery present in prokaryotes.

## Introduction

“The immediate utility of an organic structure often says nothing at all about the reason for its being.”—Richard Lewontin and Stephen Jay Gould [1]

The term RNA interference (RNAi) refers to a range of molecular processes that use a small RNA fragment as a guide to target specific nucleic acid sequences and regulate gene expression [2]. In animals, these processes are grouped into 3 major categories, depending on the origin of the small RNA: the microRNA (miRNA) pathway, the small interfering RNA (siRNA) pathway, and the Piwi-interacting RNA (piRNA) pathway [3]. However, these categories are often blurred, owing to a high degree of cross-talk between the 3 pathways [4–6]. Of the categories of RNAi processes, it is generally agreed that the siRNA pathway is the most ancient [7,8]. The prevailing view is that RNAi evolved as a defense response against transposable elements (TEs) and RNA viruses in eukaryotes [2,7–9]. An alternative view has been proposed, in which the basic RNAi machinery may have evolved to favor heterochromatin formation and centromeric assembly in eukaryotic chromosomes [10]. However, despite the extensive conservation of these chromosomal functions among eukaryotes, the defense-based hypothesis is still favored by most [11].

**Fig 1:**
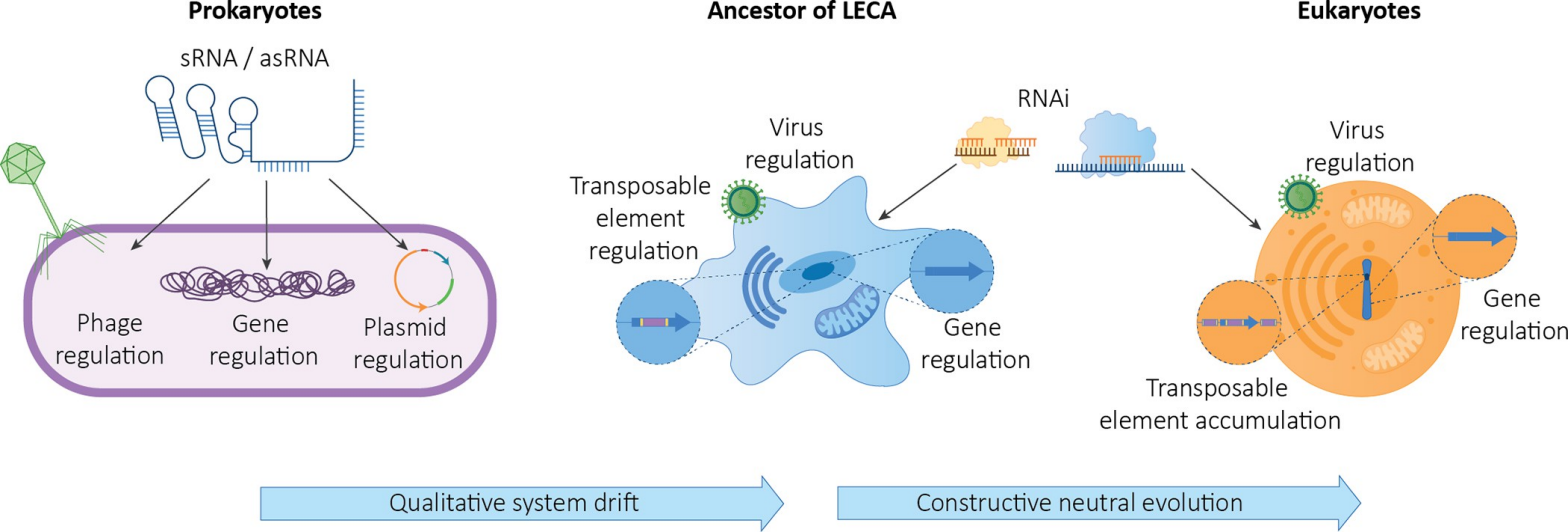
Evolution from prokaryotic RNA-mediated gene regulation to eukaryotic RNAi. We propose that the evolutionary journey from prokaryotic RNA-mediated gene regulation to eukaryotic RNAi comprised 2 distinct evolutionary process. The first involved changes in the molecular machinery without changes in the final outcomes of the process (qualitative system drift). The second involved a ratcheting cascade caused by the suppressive role of RNAi on the deleterious effects of TEs, as postulated by CNE. asRNA, antisense RNA; CNE, constructive neutral evolution; LECA, last common eukaryotic ancestor; RNAi, RNA interference; sRNA, small RNA; TE, transposable element.

Integrating a range of viewpoints is becoming increasingly viewed as necessary to understand complex biological phenomena [12,13]. Although the siRNA pathway often has a role in defense, it cannot be reduced conceptually to performing only that function. Viewing RNAi systems as having roles in both defense and regulation can reveal new avenues through which to understand their evolutionary origins. Furthermore, considering the evolutionary genesis of defense-related processes as mechanisms for regulation can lead to substantially different interpretations and distinct testable hypotheses [14]. In this Essay, by combining perspectives from different fields of research, we propose a new nonadaptive hypothesis on the origin of RNAi that helps to explain the connections between regulatory and defense functions, and supports the idea that the presence of RNAi in the last eukaryotic common ancestor (LECA) may have been the cause, not a consequence, of the invasion of early eukaryotes by TEs [15]. Central to our hypothesis is that RNAi originated from an ancient and widespread prokaryotic RNA regulatory system by qualitative system drift through constructive neutral evolution (CNE). Our hypothesis is based on 2 main pillars: first, that process homology, rather than gene or protein homology, explains how different molecular machineries produced by qualitative system drift in different organisms deliver the same biological process [16–18] and second, that CNE theory, which explains how preexisting “presuppressive” activities on deleterious mutations (e.g., that buffer the harm to the cell without removing the cause) may lead to an irreversible ratchet-like cascade of events that give rise to biological complexity [19–23] (Fig 1).

We begin by discussing some shortcomings of the defense-based hypothesis. To avoid confusion, we refer to the RNAi “defensive role” as its direct effect on viruses and TEs, and to the RNAi “regulatory role” as its general activities on the host genome. However, it is worth noting that a defensive role may be a mixture of different virus-specific or TE-specific processes depending on the species, whereas the regulatory role may also have important immunological functions, such as those related with tissue repair or inflammation. This section will highlight the close interaction between genomic defense and genomic regulation.

### Shortcomings of the defense-based hypothesis

Based on phylogenetic evidence, it is generally accepted that LECA had a functional RNAi pathway composed of the core components: Dicer, an RNase III-like endonuclease that processes double-stranded RNAs (dsRNAs); Argonaute and PIWI, 2 classes of the same family of endonucleases that use sRNAs as guides; and the RNA-dependent RNA polymerase (RdRP), which catalyzes the synthesis of the dsRNA [2,7,8]. The defense-based hypothesis proposes that this ancestral RNAi system was primarily a form of defense against viruses and transposons, rather than a regulatory process. We assert that there are 4 main shortcomings on this defense-centered view, as discussed below.

#### Shortcoming 1

As several unicellular eukaryotes have lost the ancestral RNAi system, it has been proposed that it must have been dispensable for LECA. This would be incompatible with RNAi performing essential functions in LECA, including defense [7,8].

If RNAi is dispensable, why would a defensive function be more likely than a regulatory one? Furthermore, dispensability also casts questions on whether RNAi arose through an “adaptive” evolutionary process or not. Regulatory networks in eukaryotes are often redundant and prone to experience shifts [16,24] and may have originated mainly by neutral evolution [24,25]. In general, only a minority of genes are deemed essential under laboratory conditions [26]. Therefore, it is perhaps not surprising that after almost 2 billion years of evolution since LECA, several unicellular eukaryotes have dispensed with RNAi. In the case of the yeast *Saccharomyces cerevisiae*, one explanation for the loss of RNAi is that the evolution of pointed centromeres (centromeres determined by a genetic signature) made RNAi-dependent centromere formation obsolete [10,27]. Despite the presence of retrotransposons in the genome of *S*. *cerevisiae* [28], this event may have enabled loss of the RNAi core proteins. That said, the loss of RNAi in some eukaryotes does not necessarily mean that it was nonessential for LECA or its ancestors.

#### Shortcoming 2

Plasmids are generally considered parasitic elements [29,30]. Therefore, the fact that prokaryotic Argonaute (pAgo) can control their presence and replication suggests a defense-related function [2,31].

Despite some questions about whether plasmids should be thought of as “parasites” per se [32], pAgo can clearly decrease plasmid transformation efficiency, modify plasmid content, and protect against phages [33,34]. However, this primarily defense-based view of the role of pAgo has been questioned as putative regulatory roles have been uncovered in a range of bacteria [34]. For example, *Synechoccus elongatus* pAgo shows no preference for targeting plasmid versus chromosomal DNA, has no effect on plasmid maintenance, and may instead participate in the process of chromosome replication, targeting the origin and terminus of replication [35]. The principal role of pAgo in *Thermus thermophilus* also seems to be related to DNA replication, helping to disentangle the concatenated circular chromosomes in the absence of topoisomerases [36]. Furthermore, in *Clostridium butyricum*, pAgo has a defensive role against phages and plasmids, yet also targets multicopy genetic elements (ribosomal DNA operons and transposons), sites of double-strand breaks, and the region of the replication terminus, displaying a broad variety of genomic regulatory activities [33]. The fact that most pAgo homologs are predicted to be catalytically inactive [31] may be associated with their roles in the recruitment of a range of different binding partners through site-specific genome targeting by pAgo [37]. In support of this idea, it is worth noting that several eukaryotic Argonaute (eAgo) activities during transcriptional and posttranscriptional gene silencing are independent from its slicer action and that different eAgo proteins are enzymatically inactive [11,38–41]. In addition, modifying the copy number of plasmids in the cell is not just a defense process but also a form of regulation. Rapid coevolution between bacteria and hosts can depend on the modulation of plasmid copy numbers to allow bacteria to respond more quickly to environmental changes [42]. Finally, there is a positive correlation between the presence of pAgo and the number of TEs [37], suggesting that that pAgo may actually favor the colonization of prokaryotes by TEs through reducing their fitness cost.

#### Shortcoming 3

Regulatory functions of RNAi specifically rely on the presence of miRNAs that are likely to have originated independently after the divergence of animals and plants. Therefore, it is likely that the siRNA pathway, which is mainly dependent on RdRP, would originally have had a defense-based function [7,8].

The common view on the origin of miRNAs has been recently challenged and may actually predate the plant–animal divergence [43–45], and the regulatory role of siRNAs could also be older than we think. Numerous studies support a regulatory role of siRNAs during development in fruit flies, mice, nematodes, and plants [46–56]. Furthermore, the extent of regulatory roles for RNAi may be underestimated given that in several basal metazoans, endogenous siRNAs frequently map to coding genes [5,57,58]. In many unicellular fungi, the siRNA pathway regulates the expression of endogenous genes, participates in stress responses, and is important in the formation of heterochromatin (reviewed in [59]). For example, in *Schizosaccharomyces pombe*, the primary role of the siRNA pathway is the formation of heterochromatin for centromere determination and gene regulation, whereas TEs are mainly eliminated through an RNAi-independent process [60,61]. In 2 phylogenetically distant protists [62], the canonical siRNA pathway regulates phenotypic variation, through posttranscriptional gene silencing in *Giardia lamblia* [63] and transcriptional gene silencing in *Paramecium tetraurelia* [64,65]. In *Paramecium bursaria*, the siRNA pathway is also important for the maintenance of a symbiotic relationship with the green algae *Chlorella*, through a process called RNA collision, which may have profound implications for the evolution of endosymbiosis, predatory behavior, and avoidance of cannibalism in early eukaryotes [66]. In the ciliate *Oxytricha trifallax*, a special Dicer-dependent siRNA pathway has a pivotal role in dosage compensation and the maintenance of chromosome copy number [67]. In ciliates such as *Paramecium* and *Tetrahymena* there is an additional, ciliate-specific, RNAi pathway that physically removes TEs during the formation of the macronucleus [68]. However, this activity is not strictly associated with the elimination of active TEs, as the system targets all forms of repetitive DNA and, in some cases, even genes [68–70]. Similarly, in the nematode *Caenorhabditis elegans*, WAGO-dependent 22G-siRNAs indiscriminately silence TEs, pseudogenes, certain genes, and other aberrant transcripts [71]. Curiously, in the ciliates *Oxytricha* and *Stylonychia*, the RNAi system uses a special group of 27-nucleotide-long small RNAs to select and protect the coding regions of the genome during the formation of the macronucleus [69,72], reminiscent of Argonaute CSR-1-dependent 22G-siRNAs in *C*. *elegans* that indirectly favor the holocentromere organization by targeting euchromatin [73,74]. Thus, many RNAi systems are apparently only indirectly involved in the suppression of parasitic elements and instead have a primary role in the maintenance of genome architecture and stability, an activity important for phenotypic plasticity and evolution in protists [15,75]. While a role for RNAi in repressing parasitic elements is not in dispute, we stress that the activities of these systems should not be viewed simply as a defense system that has evolved to discern elements of self from nonself. Indeed, sometimes the difference between genome defense and gene regulation is purely semantic, especially in the case of domesticated TEs [76–78].

#### Shortcoming 4

The patterns of RNAi-mediated gene regulation are so diversified and poorly conserved among eukaryotes that they are probably lineage-specific. By contrast, the defense function of RNAi against TEs and viruses is broadly conserved and constitutes strong evidence to infer its evolutionary origin [2,7,8,10,57].

Identifying the most conserved role of a given process among a range of taxa may suggest the most parsimonious hypothesis about its origin and function, but not necessarily the correct one, as can occur with exaptation, when a trait’s function switches through evolution [79].

Moreover, the proposed antiviral defensive role of RNAi seems poorly conserved across taxa. Recent studies failed to find evidence of an antiviral effect of RNAi in several basal metazoans, suggesting that it was potentially absent in the common ancestor of earthworms, sponges, and sea anemones and might be a later acquisition in metazoa [80]. Interestingly, the most conserved role of the RNAi system among eukaryotes is in the control of heterochromatin formation, modeling genome architecture, maintaining genomic stability, and enabling centromere determination [10,11,15,27,54,73,81–88].

Could the loss of RNAi in several unicellular eukaryotes relate to their small genome size and low complexity, meaning that the RNAi-dependent mechanisms were redundant [7]? In the next section, we look at how this might also have been the case with LECA.

### Transposable elements and the last eukaryotic common ancestor

Compared with prokaryotic genomes, the genomes of eukaryotes contain many more TEs [88,89]. Therefore, the idea that eukaryotes experienced a massive TE invasion and developed RNAi as a defense process to control them is appealing [15,88,90,91]. According to the defense-based hypothesis, this evolutionary event should have occurred in the ancestor of LECA, as RNAi was likely already present in LECA [7,8]. However, given that the hypothesis also notes that RNAi is dispensable in LECA and other unicellular organisms [7,8], by definition any defensive role cannot have been essential for survival. We may resolve this shortcoming by assuming that RNAi was present and functional in LECA but become fundamental only later in evolution. However, this assumption raises some questions. For example, why should the origin of RNAi have been “adaptive” if it was dispensable at the beginning, and why did TEs proliferate despite the presence of RNAi? In this section, we aim to answer these questions and explore the causal connection between RNAi and TEs.

An increase in genome size in eukaryotes with a small effective population size is predicted by the laws of population genetics [25,88]. Decreasing effective population size, and thus decreasing the power of natural selection to maintain an optimized genome, renders genetic drift the predominant evolutionary process driving genome evolution. Known as a “drift barrier,” this phenomenon generally results in a performance reduction of biological traits [92]. For eukaryotes with a small effective population size, unless there are constraints that favor the selection for small genomes [93,94], it is not possible to maintain the same low genome size found in ancestors with larger effective population sizes [25,88]. For this reason, there is a trend toward accumulation of TEs in eukaryotic genomes (especially in metazoans). There is also a concomitant increase in the number of introns, pseudogenes, and repetitive elements [89,95]. However, this trend is likely the result of an “insertion bias” (i.e., a trend toward accumulation of genomic sequences like pseudogenes and TEs) in eukaryotes, whereas the effect of genetic drift in prokaryotes is a reduction in genome size [96]. In summary, the amount of TEs integrated into a genome is the result of a combination of natural selection, genetic drift, and insertion and/or deletion biases [96].

There is a threshold in genome size (and an inversely correlated effective population size) that allows TE persistence. Near or below this threshold, TEs struggle to maintain themselves in a population [88,97]. Unfortunately, determining whether LECA had a small or large population size is problematic [98]. Based on comparative genome analyses of several eukaryotic supergroups, we can infer that LECA’s genome complexity should have been more or less comparable with that of an extant free-living unicellular eukaryote [99], which lies near or below this TE persistence threshold [88,97]. Therefore, LECA should not have struggled with TEs and, consequently, should not have been subjected to selective pressure to evolve or maintain a new defensive system. This is exemplified by unicellular eukaryotes that lack RNAi [7].

It is possible that the effective population size of LECA or its ancestor was smaller than anticipated. Or that physiological conditions changed such that LECA needed to evolve a defensive RNAi system. For example, selfish elements such as plasmids in prokaryotes are often self-regulated. For their maintenance, it is important to support a rate of replication that does not compromise survival of the host but is also not so low as to risk extinction [100,101]. For the same reasons, prokaryotic TEs are often self-regulated [101–103]. Whereas population genetics studies predict that self-regulated TEs are likely to emerge in bacteria, conditions in organisms with relatively free recombination, such as LECA, are more restrictive [104]. In diploid eukaryotes, only dominant lethal or sterile mutations associated with transposition count as a driving force for selection of a repressor [104]. These conditions may have favored the evolution of RNAi for defense in the ancestor of LECA. Although we cannot formally exclude this possibility, we think it is unlikely for 4 reasons. First, ectopic recombination and purifying selection are the main factors in controlling TEs [15,105,106] and should have maintained TE copy number in the ancestor of LECA. By comparison, RNAi-deficient fungi of the Cryptococcus genus have shorter centromeres and a concomitant loss of full-length retroelements [82]. Second, tolerance toward TEs may be selected in the absence of RNAi silencing, as was reported for the case of p-elements (a class of TEs) in *Drosophila* [107]. Third, if the ancestor of LECA had no defense against selfish elements or RNA viruses, it is not clear how it survived in a bacteria-dominated world [98,108] before evolving an entirely new and complex molecular process. And fourth, although not as common as in prokaryotes, the conditions for the selection of self-regulated TE can also occur in eukaryotes [104,109,110]. In short, the ancestor of LECA did not need to evolve a new molecular process to control TEs; natural selection, ectopic recombination, molecular machineries inherited from prokaryotes, and the presence of self-regulated TEs should have been sufficient.

As there was potentially no strong selective pressure to maintain a new defensive system (i.e., it was dispensable) [7], it is possible that the RNAi defense function, and possibly even the regulatory one, may have originated through neutral events. For example, the presence of RNAi would likely have reduced ectopic recombination [15,105], favoring TE integrity and accumulation in centromeric regions that are protected by heterochromatin-mediated silencing [15,105,106,111]. It would also have attenuated the deleterious effect of TEs, increasing the probability of their fixation in the population [112]. Therefore, rather than a defense against TE invasion, RNAi may actually be one of the causes of TE proliferation in eukaryotes [15].

### The evolution of RNAi through a CNE lens

**Fig 2:**
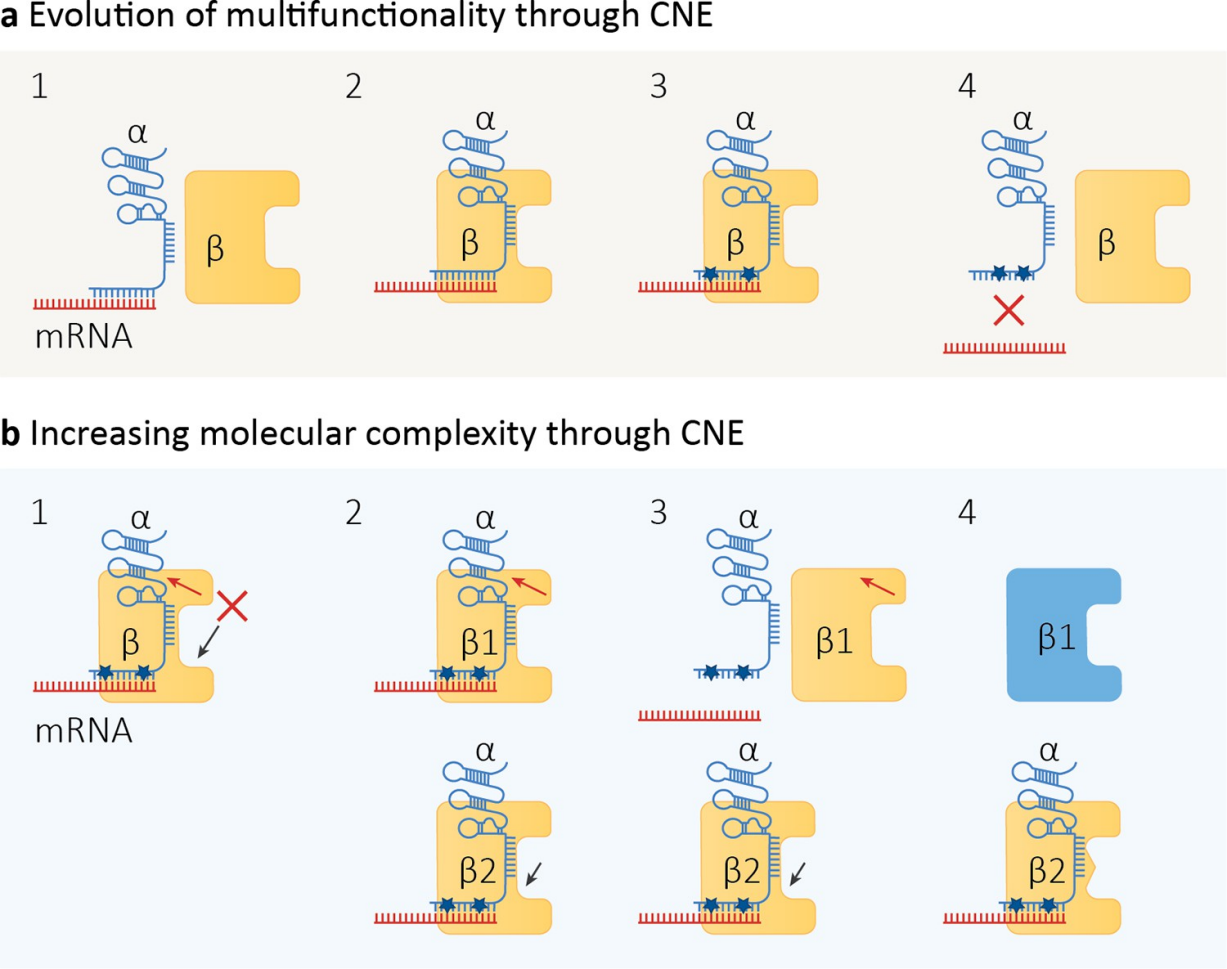
General examples of CNE. (**a**) Multifunctionality evolution through CNE. (1) α is a generic noncoding RNA that mediates RNA silencing on its target mRNA (red), and β is an enzyme that participates in cellular metabolism. (2) By chance, α and β may interact in the cellular environment creating an ephemeral complex. (3) Excess capacity in β results in the stabilization of the α–mRNA complex and then exerts a suppressive effect on mutations in α, which are now no longer deleterious [19,114]. (4) At this stage, α is reliant on interaction with β to exert its activity, while β has gained a new function in becoming a chaperone for α. (**b**) Increasing molecular complexity by CNE. (1) Mutations that inhibit the catalytic function of β (black arrow) or compromise α–β interactions (red arrow) are dangerous for the organism and would be eliminated by purifying selection. (2) In the case of gene duplication of β (represented by β1 and β2), there is now an excess capacity in the system that can exert a presuppressive activity [19,113]. (3) Mutations that compromise the stability of α–β interactions in β1 and the enzymatic activity in β2 are no longer deleterious since that function can be carried out by the other protein. (4) β1 and β2 can now evolve as 2 different proteins, without adaptive evolution. CNE, constructive neutral evolution.

Is it more likely that RNAi evolved as a defense against parasitic genetic elements at the cost of disrupting or altering gene regulation, or as a multifunctional process that can regulate gene expression, favor heterochromatin formation, and inhibit TEs and viruses? When addressing this fundamental question, one should bear in mind that biological complexity can emerge by neutral events and may become essential even if it is useless [19,22,113–116]; that a specific function can originate from exaptation of a trait that was selected for a different function [79]; and that the characteristics of most biological entities may produce important physiological and evolutionary by-products [1]. Furthermore, macromolecules are also dynamically interconnected inside the crowded cellular environment [21], with an incredible number of possible interactions and activities, most of which will have no effect on fitness [19,115]. This is what Arlin Stoltzfus calls “excess capacity” [19,20]. Excess capacity suggests that it is impossible to prestate all the possible activities of a given biological entity (such as an enzyme), as the nature and number of these activities are heavily dependent on the specific history and context of the entity in question [117]. Excess capacity is key to understanding how neutral evolution can build cellular complexity, as described by the CNE theory [19], which posits that potentially dangerous effects, such as a deleterious mutation, can be rendered innocuous by preexisting conditions owing to the excess capacity of a protein or a biological structure, thereby allowing the mutation to accumulate and leading to an irreversible cascade of events [21] (Fig 2).

In recent years, CNE has been used to explain the evolution of several molecular processes, biological structures and genomic features, including scrambled genes in ciliates, RNA editing, multimeric protein formation, the spliceosome, and the ribosome [19,21–23,118–121]. For example, CNE can explain the presence of cryptogenes in the mitochondrial genome of *Trypanosoma*. In the production of a functional protein, the mRNA of these cryptogenes undergoes substantial RNA editing [122]. It is unlikely that this molecular process could have evolved to correct dysfunctional genetic sequences [19,119]. Therefore, the predisposition for RNA editing must have been present in the cell as excess capacity before the first deletion appeared. With a process that can tolerate and fix deleterious mutations, there is much less purifying selection acting on the genome, producing a ratchet-like cascade of events [21]. RNA editing was therefore not the solution, but the cause of cryptogenes [19,119].

CNE theory can be applied to the RNAi system [21]. If the ancestor of LECA experienced an invasion of transposons and RNA viruses, it likely could not have survived until the evolution of an entire new molecular process occurred. Therefore, the ancestor of LECA must already have had systems in place to control transposons and viruses [10], and the presence of an RNAi system that promoted tolerance toward TEs and RNA viruses, by reducing their fitness cost [112], may actually be the cause, rather than a consequence, of the transposon invasion [15].

In the rest of this section, we propose a step-by-step route by which molecular system drift from prokaryotic RNA antisense regulation could have led to eukaryotic RNAi through CNE, and how this affected genome evolution.

### The origins of RNAi

Although it is generally assumed that dsRNA is a hallmark of nonself, antisense transcription and dsRNAs are widespread in prokaryotes [123–126]. As such, dsRNA cannot be associated exclusively with nonself, and proteins or processes acting on dsRNAs are not necessarily entities responding to a foreign pathogen; they may alternatively carry out functions of physiological importance for the host, and/or respond to improper transcription. Why then is the accumulation of dsRNA sometimes perceived as a pathogen-associated molecular pattern in prokaryotes and eukaryotes? One possible answer comes from the discontinuity theory of immunity [127]. Biological systems react to sudden abnormal changes in their intracellular and extracellular context [127]. Consequently, when viral replication produces a rapid increase in the amount of dsRNA, it activates an immune response. This phenomenon can be understood as the response to an anomaly, independently of its origin (i.e., self or nonself). The focus here is on the rapid accumulation of dsRNAs or RNAs without proper chemical secondary modification, not on the source that produces them.

Long before the discovery of RNAi, antisense RNA regulation was considered an ancient and widespread form of gene regulation in prokaryotes [100,101,128]. These antisense RNAs are classified as *cis*-acting antisense RNAs (asRNAs) when they are produced close to the gene or the structure that they regulate (they are natural antisense transcripts) and when they work with perfect complementarity or as *trans*-acting small RNAs (sRNAs) when they do not need perfect complementarity and can work in trans, often in association with chaperones that increase the affinity for their target [123,124,129]. This form of regulation promotes the degradation of dsRNA by the action of RNase III (a dsRNA-specific endonuclease) and the production of small dsRNAs [130], or by blocking or inducing translation [131]. This kind of RNA regulation controls a wide range of activities inside cells, such as riboswitch elements, toxin–antitoxin modules, mobility, DNA repair, metabolism, gene regulation, cell shape, and biofilm formation [101,123–125,129,132]. It is also used by plasmids, TEs, and phages for self-regulation [100,101,128]. It has been calculated that the action of RNase III alone may regulate approximately 10% of protein expression levels in *Escherichia coli* [133]. Thus, the use of dsRNA may well be an ancestral regulatory process.

#### Qualitative system drift

**Fig 3:**
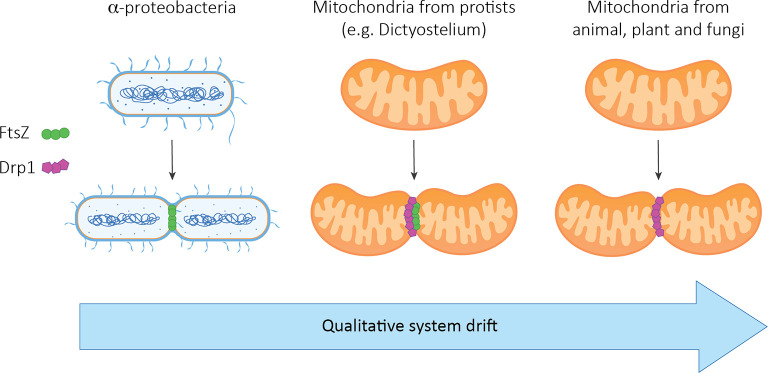
An example of qualitative system drift during binary fission. Mitochondria in the protist *Dictyostelium*, retain the bacterial protein FtsZ together with the eukaryotic Drp1, whereas in mitochondria in higher eukaryotes, only Drp1 is present, indicating a shift in the molecular effector for binary fission during evolution.

Despite its analogies with RNAi, it was assumed that it is unlikely that the siRNA pathway could have evolved from the prokaryotic antisense regulatory process owing to the lack of homology between the protein machineries [8]. However, a lack of homology between the components of 2 processes does not necessary imply a lack of homology between the 2 processes themselves [16,17]. One notable example is the phenomenon of prokaryotic cell division by binary fission. Several cellular processes and proteins participate in cytokinesis, among them the tubulin homolog FtsZ has a crucial role [134]. Mitochondria (which are organelles derived from α-proteobacteria endosymbionts) in some protists retain an FtsZ homolog, together with the eukaryotic dynamin family protein Drp1, whereas mitochondria in animals, land plants and fungi rely solely on Drp1 [134–136]. As such, during evolution there has been a shift in the molecular effector for binary fission (Fig 3).

Selection acts at the level of the phenotype, not the genotype, and these 2 hierarchical levels are evolutionarily dissociable [16,17,137–139]. Therefore, molecular processes can undergo dramatic shifts in their qualitative (and quantitative) composition, without necessarily altering the phenotypic outcome, an event also predicted by population genetic studies [24,137,138,140,141]. Various names have been coined to address this phenomenon, the most common terminologies being “developmental system drift” [142,143], “phenogenetic drift” [137], or “qualitative and quantitative system drift” [138,140].

#### Qualitative system drift through CNE

How may such qualitative system drift have occurred to move from prokaryotic RNA regulation to eukaryotic RNAi? Although it is impossible to know the correct answer, we can hypothesize on the basis of studies of gene regulation in extant organisms. For example, in organisms that lack an RdRp, such as fruit flies and mice, regulatory siRNA can originate from naturally occurring dsRNAs [49,51,52]. Consequently, the generation of the first proto-siRNAs may have derived from the degradation of naturally occurring dsRNAs. In prokaryotes, pervasive transcription and gene regulation by asRNA result in the accumulation of short dsRNAs through the activity of RNAse III [123,130]. In the overcrowded cellular milieu, these short RNAs would be likely to interact with a range of components in stable and unstable ways [21]. One of these components could have been a pAgo, which are present in about one-third of sequenced archaeal genomes and in 10% of bacterial genomes [2,31] and represent a class of extremely versatile proteins that can bind 5′-phosphorlylated short RNAs and DNAs or 5′-hydroxyl RNAs and use them to target both DNA or RNA [144–146].

**Fig 4:**
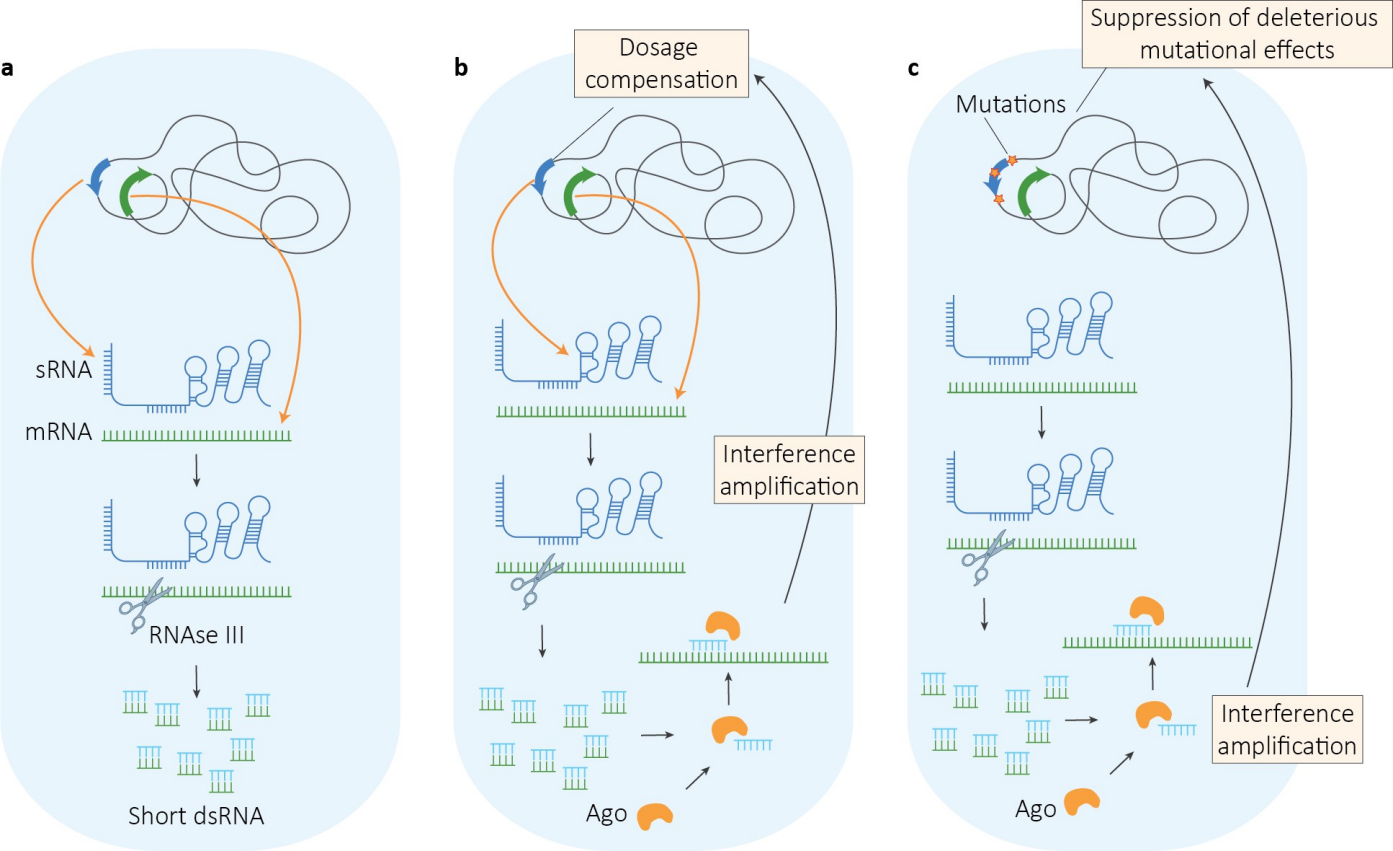
Hypothetical transition from bacterial asRNA gene regulation to eukaryotic RNAi due to CNE. (**a**) asRNAs (not in figure) or sRNAs may lead to the production of short dsRNAs. (**b**) Prior to degradation, the short dsRNAs may interact with cellular proteins such as Ago and become guide RNAs. The slicer activity of Ago may cause an amplification of the interference effect that triggers the down-regulation of the asRNA or sRNA by dosage compensation. (**c**) Mutations that reduce the expression or functionality of the asRNA or sRNA are now likely to appear and accumulate owing to the suppressive effect of Ago, causing irreversibility to this process. Ago, Argonaute; asRNA, antisense RNA; CNE, constructive neutral evolution; dsRNA, double-stranded RNA; RNAi, RNA interference; sRNA, small RNA.

In some eukaryotes ancestors of LECA, the interaction between Argonaute and these short RNAs derived from the degradation of dsRNAs may have led to an amplification of the interference phenomenon carried out by asRNAs or sRNAs (Fig 4A). During this stage, this excess capacity was likely to be redundant in the best case scenario, and detrimental for the cell in the worst. However, intracellular processes do not work in isolation [21,147,148], hence, gene down-regulation and related phenomena of dosage compensation are likely to arise [147]. Therefore, the amplification of the signal due to Argonaute might have induced the down-regulation of the sRNAs (Fig 4B). At this point, mutations that permanently reduce the expression of these genes are no longer dangerous, and they become more likely to appear and get fixed in a population. Therefore, the action of Argonaute on the interference signal becomes essential to maintain an appropriate level of regulation and can no longer be reverted (Fig 4C). In addition, mutations in the RNA loop that affect the functionality of the sRNA are likely to accumulate, as the silencing effect is now mediated by Argonaute. For example, in some cases, translational repression induced by sRNAs is sufficient for gene silencing [149], and the sRNA decay can be mediated by RNase III producing short RNAs [150]. The interaction of Argonaute with these short RNAs can amplify the interference signal and, consequently, decrease the importance of translational repression. This would lead to the accumulation of mutations in the sequence of the sRNA involved in inhibiting translation, as now they are no more harmful, making the presence of Argonaute essential and irreversible. These are some examples of suppressive effects on deleterious mutations and the consequent ratcheting cascade that characterize CNE [19,21,119,121]. The evolution of Dicer, and the acquisition of RdRp respectively, may have further expanded the specificity and possibility of the ancestral RNAi system. For example, with RdRp, it is possible to produce dsRNA that acts directly on mRNAs that must be controlled without natural antisense transcripts, as observed for the regulated phenotypic variation in the protists *G*. *lamblia* and *P*. *tetraurelia* [63–65].

Through such a transition, the eukaryotic RNAi system would have been able to take over the physiological effects of prokaryotic interference, including the ability to control TE and virus replication. In this scenario, no specific function was selected. Instead, qualitative system drift occurred that changed the molecular components and processes without changing the regulatory outcomes of the process. This transition may have been neutral, but the long-term evolutionary consequences would not have been. As with all processes originated by CNE, RNAi systems forced eukaryotes on an irreversible evolutionary trajectory that has shaped their genome architecture ever since [15,82] (Fig 1) and engendered a tight relationship between genome regulation and genome defense.

#### Link between sRNAs and RNAi

The connection between sRNAs in prokaryotes and organelles on the one hand, and eukaryotic RNAi on the other, may provide support for this hypothesis. In the thale cress *Arabidopsis thaliana*, 25% of the transfer RNA (tRNA)-derived sRNAs that immunoprecipitate with Argonaute-1 come from plastid tRNAs, suggesting the presence of a retrograde signaling pathway [151]. In humans, a mitochondrial sRNA derived from the polycistronic mitochondrial RNA can interact with Argonaute-2 and possibly target the 3′-UTR region of *CFLAR* [152]. The intracellular pathogen *Mycobacterium marinum* produces an sRNA where the secondary structure is processed like an miRNA by the host cells, and which interacts with the RNA-induced silencing complex [153]. Notably, bacterial sRNAs and asRNAs are characterized by a complex secondary structure with several loops [100,101]. These structures may have been the source of small dsRNAs for a proto-RNAi pathway, in which siRNAs or miRNA-like RNAs were produced using a similar mechanism to *Drosophila*, where hairpin RNAs are processed to produce siRNAs [50].

Another example of the connection between prokaryotic sRNA and RNAi is given by small nucleolar RNAs (snoRNAs). snoRNAs are a widespread class of eukaryotic small RNA with an archaeal origin and can carry out a broad variety of functions, including posttranscriptional RNA modification [154–156]. Owing to the fact that some snoRNAs can interact with the core proteins of the RNAi system and give rise to miRNA-like RNAs in animals, plants, protists, and yeasts, they have been proposed to have an ancient link with RNAi [154, 155]. Interestingly, in *Drosophila melanogaster*, there is evidence of cross-talk between the miRNA and siRNA pathways during biogenesis of snoRNA-derived small RNAs [154]. Furthermore, another class of ancient small RNAs has been recently proposed as a link between prokaryotic antisense RNA regulation and eukaryotic RNAi: the tRNA-derived sRNAs [157,158]. The evolutionary model proposed in this Essay is also compatible with this new hypothesis.

Crucially, as an evolutionary process, molecular system drift of RNAi is still in action. For example, in budding yeast (which did not lose its RNAi system), Dicer is replaced by another enzyme that evolved from the RNAse III RNT1 (confusingly named DCR1) [159]. Other similar examples are the noncanonical Dicer of the protist *Entamoeba hystolytica* [160] and Dicer-independent siRNAs in the fungus *Neurospora crassa* [161].

## Conclusions

Gene regulation based on noncoding RNA is ancient and might even precede the origin of cellular life [2,162,163]. It was proposed by François Jacob and Jacques Monod decades before it could be conclusively demonstrated [164] and subsequently became well studied during the 1980s [101]. The discovery of eukaryotic RNAi at the end of the 1990s represented a biotechnological revolution [165]. However, it did not revolutionize our understanding of gene regulation or genomic defense since it represented only a variant (albeit a new and fascinating one) of an ancient and successful regulatory process: the use of antisense RNAs to regulate gene expression and parasitic element replication. Importantly, RNAi is not the only process that straddles regulatory and defensive roles; DNA methylation shows the same characteristic [15,166]. Indeed, it has been debated whether DNA methylation evolved as a genome defense process or as a regulatory process in invertebrates [167]. Another example is the CRISPR/Cas system in prokaryotes, which is generally considered a defense system but can have also important genome repair and regulatory functions [168].

These examples highlight the complexity of biological processes and the importance of combining different perspectives to apply the best research methodology to understand them [12,13,114].

Eukaryotic regulatory processes have indeed complex architectures, to the point that they have been described as “baroque structures” [24] or “Rube Goldberg machines” in comparison with the prokaryotic ones [169]. To understand this complexity, we do not necessarily need to search for adaptive explanations as the effect of neutral evolution can be sufficient [19,24,25,113,114]. On the same basis, regardless of any selective advantage, we should also expect a progressive increase in complexity for antisense RNA regulation in eukaryotes compared with prokaryotes, with the evolution of new regulatory elements and protein duplication and subfunctionalization (Fig 2B). In support of this idea, it is difficult to envisage an adaptive explanation for the origins of the 19 different functional Argonaute proteins reported by [170] in *C*. *elegans*, an organism composed of 959 cells (1,031 in males) and with a genome size 30 times smaller than that of humans.

In this Essay, we propose a step-by-step route which explains “how” RNAi could have emerged, without adaptive driving forces, from the molecular machineries present in the ancestor of the LECA through a qualitative system drift caused by CNE. We expect that our model can help to understand eukaryotic genome architecture evolution and the relationship between genome regulation and genome defense.

## Abbreviations:

asRNA: antisense RNA
CNE: constructive neutral evolution
dsRNA: double-stranded RNA
eAgo: eukaryotic Argonaute
LECA: last eukaryotic common ancestor
miRNA: microRNA
pAgo: prokaryotic Argonaute
piRNA: Piwi-interacting RNA
RdRp: RNA-dependent RNA polymerase
RNAi: RNA interference
siRNA: small interfering RNA
snoRNA: small nucleolar RNA
sRNA: small RNA
TE: transposable element
